# The Significance of *RHD* Genotyping and Characteristic Analysis in Chinese RhD Variant Individuals

**DOI:** 10.3389/fimmu.2021.755661

**Published:** 2021-11-12

**Authors:** Yanling Ying, Jingjing Zhang, Xiaozhen Hong, Xianguo Xu, Ji He, Faming Zhu

**Affiliations:** ^1^ Blood Center of Zhejiang Province, Institute of Transfusion Medicine, Hangzhou, China; ^2^ Key Laboratory of Blood Safety Research of Zhejiang Province, Hangzhou, China

**Keywords:** RhD variant, *RHD* allele diversity, genotype, molecular mechanism, bioinformatics

## Abstract

**Background:**

RhD is the most important and complex blood group system because of its highly polymorphic and immunogenic nature. RhD variants can induce immune response by allogeneic transfusion, organ transplantation, and fetal immunity. The transfusion strategies are different for RhD variants formed by various alleles. Therefore, extensive investigation of the molecular mechanism underlying RhD variants is critical for preventing immune-related blood transfusion reactions and fetal immunity.

**Methods:**

RhD variants were collected from donors and patients in Zhejiang Province, China. The phenotypes were classified using the serologic method. The full coding regions of *RHD* gene were analyzed using the PCR-SBT method. The multiplex ligation-dependent probe amplification (MLPA) assay was used to analyze the genotype and gene copy number. SWISS-MODLE and PyMOL software were used to analyze 3D structures of RhD caused by the variant alleles. The effect of non-synonymous substitutions was predicted using Polymorphism Phenotyping algorithm (PolyPhen-2), Sorting Intolerant From Tolerant (SIFT), and Protein Variation Effect Analyzer (PROVEAN) software.

**Results:**

In the collected RhD variants, 28 distinct *RHD* variant alleles were identified, including three novel variant alleles. RH-MLPA assay is advantageous for determining the copy number of *RHD* gene. 3D homology modeling predicted that protein conformation was disrupted and may explain RhD epitope differential expression. A total of 14 non-synonymous mutations were determined to be detrimental to the protein structure.

**Discussion:**

We revealed the diversity of *RHD* alleles present in eastern Chinese RhD variants. The bioinformatics of these variant alleles extended our knowledge of RhD variants, which was crucial for evaluating their impact to guide transfusion support and avoid immune-related blood transfusion reactions.

## Introduction

The Rh blood group system plays a pivotal role in the field of blood transfusion and severe hemolytic disease of fetus/newborn due to its high polymorphism and strong immunogenicity (1–3). Except for the common RhD-positive and -negative phenotypes, populations exhibit numerous variants, including weak D, partial D, and Del types. The differential expression of antigen on the surface of red cells caused by RhD variants may lead to a corresponding immune response during allogeneic transfusion therapy, resulting in a series of serious consequences such as severe hemolytic transfusion reaction and organ transplant rejection. In clinical settings, it usually relied on the phenotypic outcomes of conventional *RHD* typing. However, most RhD variants could not be precisely identified using the serologic method, imposing great dangers to the immune response caused by RhD variant blood transfusion. RhD-negative blood recipients may develop anti-D antibodies following transfusion of some RhD variant red blood cells in practice (4–6). The RhD variants genotyping is greatly significant for avoiding immune-related blood transfusion reactions.

Most RhD variants could be traced back to DNA variation, including single nucleotide variation (SNV), multiple nucleotide variants (MNV), base insertions or deletions, and hybrid allele (7-9). So far, over 400 *RHD* alleles have been registered and nominated by the International Society of Blood Transfusion (ISBT), which were divided into four categories (https://www.isbtweb.org/working-parties/red-cell-immunogenetics-and-blood-group-terminology). The genetic alterations of *RHD* alleles differentially influence RhD epitope attributes and quantity expression, resulting in various variants with potentially distinct immune responses. However, due to a lack of these agents for all immunogenic epitopes in the laboratory, the serological phenotypic detection of RhD variants is often unclear. Notably, the ability to test antigens without using serologic reagents is a major medical achievement, which is beneficial for locating compatible donor units and can be life-saving. As a result, it is urgent and necessary to perform *RHD* genotyping and determine the phenotypes of RhD variants in practice.

Current research on the distribution of variant *RHD* alleles has been mostly focused on European donor and patient cohorts (10–14). Additionally, there are some corresponding reports on the diversity of *RHD* alleles in African and Asian populations (15). The knowledge about overall characteristics of RhD variants in China remains relatively limited. We aimed to obtain further knowledge of RhD variant genotype and explore the genetic resources of *RHD* gene in the Chinese population based on the diversity of genetic characteristics in different populations and allele-specific antigen and antibody immune response. Herein, RhD variants collected from a specific period were tested using different typing assays for *RHD* allele sequence and copy number variation analysis. Moreover, bioinformatics analysis was processed *in silico*, which could enhance our understanding of the influence of genetic variants on antigen differential expression and their implications for immune-related transfusion reactions and fetal immunity.

## Materials and Methods

### Study Specimens

The study specimens are collected in daily work. These samples were first suspected to be RhD variants or anti-D negative and were sent to our reference laboratory for further identification. Portions of them were drawn from blood donors in Blood services in the Zhejiang Province, while the remainders were drawn from patients in hospitals of Hangzhou City in Zhejiang Province, China. Zhejiang Province is located in eastern China. These individuals provided informed consent, and the research was approved by the ethics committee of Blood Center of Zhejiang Province. Genomic DNAs were extracted using a commercial MagDNA pure LC DNA isolation kit (RBC Bioscience Corporation, Taiwan, China) according to the manufacturer’s instructions.

### Serological Test

RhD phenotypes were determined using conventional serological kits. Using microplate testing, blood donors were selected from regular preliminary screening of RhD types by anti-D reagent (IgM, Clone LDM1, Alba Bioscience Ltd, UK). RhD preliminary screenings of negative or weak expression blood donors and patient samples from hospital were further identified using a tube test (IgM/IgG blend, Clone TH-28, and MS-26, Merck Millipore Ltd, Livingston, UK). Agglutination reactions were graded on a scale of 0 to 4+, and phenotypes were classified as RhD negative (0), weak RhD (±, 1+, and 2+), or RhD positive (3+ and 4+). RhD-negative and weak RhD specimens were further determined by indirect agglutination test (IAT) using two of the following anti-D reagents according to reagent supply (1-IgM/IgG blend, Clone RS1126, and MS-26, Lorne Laboratories Ltd, Twyford, UK; 2-IgM/IgG blend, Clone P3X61, P3X21223B10, P3X290, and P3X35, Diagast Ltd, Loos, France; 3-IgM/IgG blend, Clone TH-28, and MS-26, Merck Millipore Ltd, Livingston, UK; 4-IgG blend, clone MS-26, Shanghai Blood Biomedicine Co., Ltd. Shanghai, China). RhD variants were defined as RBCs that agglutinate with anti-D reagent but had agglutination intensity of less than 2+, or agglutinated with only a few anti-D reagents, or were not agglutinated by the tube method but reacted with anti-D by IAT.

### *RHD* Genotyping Using PCR-SBT

PCR was used to amplify the sequences of 10 exons of *RHD* gene. The amplification and sequencing primers were selected according to our prior report (16). The amplification conditions were optimized to the same reaction program as follows: 95°C 5 min; 95°C 30 s, 64°C 30 s, 72°C 90 s, 30 cycles; 72°C 10 min. The content of reaction system is identical to that described previously (16), except that DNA polymerase was changed to 0.5 U La-Taq (TaKaRa, Dalian, China).

### Heterozygosity Detection Using Hybridization Box Test

*RHD* zygosities of all RhD variants were analyzed by detecting hybrid Rhesus box using PCR-SSP method described previously (17).

### Genotype and Copy Number Analysis Using MLPA Method

The copy number of *RHD* gene was analyzed by the RH-MLPA method using a commercial blood group genotyping kit (MRC-Holland, Amsterdam, the Netherlands), which includes three sets of MLPA probe mixtures P401, P402, and P403, as well as reaction system reagents. The three sets of probes contain 44 *RH* allele wild-type and mutant probes. The presence and proportion of probe signals were utilized to determine the existence of wild-type and/or variant alleles as well as the corresponding copy number (18). MLPA operation process and data analysis were conducted following the manufacturer’s procedures.

### Protein 3D Structure Analysis

3D structure models of wild-type and prematurely terminated RhD protein were generated from the crystal structure of RhCG protein (PDB code: 3HD6) (19) template using SWISS Model (https://swissmodel.expasy.org/interactive) (20). The RhCG protein is the most similar structure to RhD, and there is yet no crystal structure for wild-type RhD. The structures of novel variant RhD proteins mutated by amino acid substitutions were analyzed using PyMOL software (21).

### The Effect of Missense Mutations Prediction *In Silico*

Polymorphism Phenotyping algorithm (PolyPhen-2) (22), Sorting Intolerant From Tolerant (SIFT) (23), and Protein Variation Effect Analyzer (PROVEAN) (24) were all employed to predict the impact of nonsynonymous substitutions on RhD protein structure.

## Results

### Preliminary Serological Results

Following preliminary RhD blood type screening and further confirmation using a tube test and ITA method, 59 specimens were designated as RhD variants for follow-up research. These samples exhibited no agglutination or weak agglutination by IgM antibody using a tube test or +~3+ agglutination strength using IAT test. Based on our results, all samples revealed similar agglutination strength in IAT test using two reagents containing IgG antibodies. Anti-D antibodies were found in the serum of two blood donors.

One case of a pregnant woman with RhD-negative phenotype was also included in this study, which was subjected to subsequent analysis after detecting anti-D antibody in the serum and obtaining as suspected result using the PCR-SSP method in the hospital.

### Genotype Distribution of D Variants

Ten exons of *RHD* gene were analyzed using the PCR-SBT method. There were 52 specimens with variant sites in the coding region. Seven specimens with no variants were identified as normal RHD*01 genotype. Another one was confirmed as an RHD-negative allele. Among them, RHD*DVI.3 homozygotes and RHD*weak partial 15 homozygotes were the two most prevalent genotypes in the Chinese population, accounting for 26.9% and 19.2% of RhD variants in this study, respectively. Two donors who produced antibodies were with the RHD*DVI.3 homozygous genotype. Using MLPA assay, nine specimens were detected as heterozygous, including heterozygosity with RHD*01, or heterozygosity for two different variant alleles. The detailed genotyping results are depicted in **Table 1**.

**Table 1:** Genotype results and the copy number determination using different assays in the Chinese RhD variants.

ID	Genotype by PCR-SBT	Genotype based on RHD-MLPA result	Copy number of RHD allele^†^	Hybrid Rhesus box results	No
1	RHD*weak partial 15	RHD*weak partial 15/-	1	*RHD+/RHD-*	10
2	RHD*weak partial 15/RHD*01EL.01	RHD*weak partial 15/RHD*01EL.01	2	*RHD+/RHD+*	1
3	RHD*01EL.01/RHD	RHD*01EL.01/RHD	2	*RHD+/RHD+*	1
4	RHD*weak partial 15/RHD*58	*/*	/	*RHD+/RHD+*	1
5	RHD*101G	*RHD/-*	1	*RHD+/RHD-*	1
6	RHD*101G/RHD	RHD/RHD*01N.04	2	*RHD+/RHD+*	1
7	RHD*01W.33	RHD*01W.33/-	1	*RHD+/RHD-*	1
8	RHD*01W.54	RHD*01W.54/-	1	*RHD+/RHD-*	1
9	RHD*01W.36	*RHD/-*	1	*RHD+/RHD-*	1
10	RHD*01W.25	*RHD/-*	1	*RHD+/RHD-*	2
11	RHD*01W.85	*RHD/-*	1	*RHD+/RHD-*	1
12	RHD*01W.47/ RHD* weak partial 15	RHD* weak partial 15/RHD	2	*RHD+/RHD+*	1
13	RHD*01W.100/RHD	RHD*01W.100/RHD	2	*RHD+/RHD+*	1
14	RHD*01W.72	*RHD/-*	1	*RHD+/RHD-*	1
15	RHD*17.01/RHD*95A	*RHD/RHD*	2	*RHD+/RHD+*	1
16	RHD*01W.1	RHD*01W.1/-	1	*RHD+/RHD-*	2
17	RHD*16.02	*RHD/-*	1	*RHD+/RHD-*	1
18	RHD*DVII.1/RHD	RHD*DVII.1/RHD*01N.04	2	*RHD+/RHD+*	1
19	RHD*01W.3	*RHD/-*	1	*RHD+/RHD-*	1
20	RHD*143G	*RHD/-*	1	*RHD+/RHD-*	1
22	RHD*782T	*RHD/-*	1	*RHD+/RHD-*	1
23	RHD*01W.122/ RHD*210_211 insG	*RHD/RHD*	2	*RHD+/RHD+*	1
24	RHD*538C/RHD*01EL.01	RHD/RHD*01EL.01	2	*RHD+/RHD+*	1
25	RHD*18A	*RHD/-*	1	*RHD+/RHD-*	1
26	RHD*D-CE(9)-D	*/*	/	*RHD+/RHD-*	1
27	RHD*03.02	*RHD/-*	1	*RHD+/RHD+*	1
28	RHD*03.03	*/*	/	*RHD+/RHD-*	1
29	RHD*06.03.01	RHD*06.03.01/-	1	*RHD+/RHD-*	14

/No available DNA for RH-MLPA assay and the copy number of RHD allele could not be calculated.

^†^The copy number of the allele was determined based on the ratio value of probe combination.

### Molecular Characterization of Variant *RHD* Alleles, Including Novel Alleles

**Figure 1:**
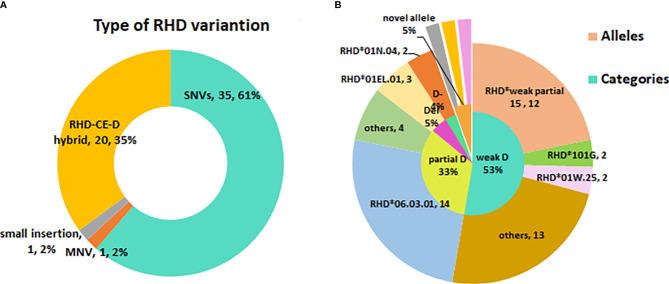
The profile of *RHD* variant allele’s diversity in eastern Chinese RhD variants. **(A)** Type of RHD variation. **(B)** The inner small circle is the allele categories map. The outer big circle is the specific allele distribution.

A total of 28 variant *RHD* alleles were identified in these RhD variants, including 20 single nucleotide variants (SNV), 1 multiple nucleotide variant (MNV), 1 insertion, and 6 RHD-CE-D hybrid alleles (**Figure 1A**). According to ISBT nomenclature classification, these variant alleles can be classified into 14 weak D alleles, 9 partial D alleles, 1 Del allele, 1 D negative allele, and 3 novel variant alleles. The number and proportion are illustrated in **Table 2** and **Figure 1B**. Among our test specimens, the most prevalent variant allele was partial D allele, RHD*DVI.3, in which exons 3–6 were exchanged with *RHCE.* Additionally, RHD*weak partial 15 alleles had a relatively high frequency. The other remaining variant alleles were rare because only one to two specimens were detected for each allele. RHD*18A is a synonymous mutation. The amino acid it encodes is located at the N-terminus of protein, which may affect transcriptional splicing of RNA. Specific allele information, including amino acid mutations, rs number, GenBank no., and membrane localization, is listed in **Table 2**.

**Table 2:** *RHD* alleles in a cohort of Chinese individuals with RhD variant phenotypes (*n* =52).

Type of RHD variation	Allele in detail	Nucleotide change	Exon location	Amino acid change	Membrane localization^†^	ISBT terminology	rs number	GenBank NO.	Allele number	Allele frequency in test (%)
RHD allele with SNV	RHD*845A	c.845G>A	E 6	p.Gly282Asp	TM5	RHD*15	rs142484009	MZ782892	12	21.1
	RHD*1227A	c.1227 G>A	E 9	p.Lys409Lys	TM12, IC7	RHD*01EL.01	rs549616139	MZ782903	3	5.3
	RHD*101G	c.101A > G	E 1	p.Tyr34Cys	EF1	*None^§^*	rs779154105	MZ782907	2	3.5
	RHD*520A	c.520G>A	E 4	p.Val174Met	TM6	RHD*01W.33	rs147421281	MZ782895	1	1.8
	RHD*365T	c.365C>T	E 3	p.Ser122Leu	TM4	RHD*01W.54	rs752408858	MZ782896	1	1.8
	RHD*842G	c.842T>G	E 6	p.Val281Gly	TM9	RHD*01W.36	/	MZ782897	1	1.8
	RHD*341A	c.341G>A	E 3	p.Arg114Gln	TM4	RHD*01W.25	s530929152	MZ782898	2	3.5
	RHD*209A	c.209G>A	E 2	p.Arg70Gln	TM2	RHD*01W.85	rs142925159	MZ782899	1	1.8
	RHD*340G	c.340C>G	E 3	p.Arg114Gly	TM4	RHD*01W.47	/	MZ782900	1	1.8
	RHD*787A	c.787G>A	E 5	p.Gly263Arg	IC5	RHD*01W.100	rs3118454	MZ782901	1	1.8
	RHD*1212A	c.1212C>A	E 9	p.Asp404Glu	IC7	RHD*01W.72	rs767611524	MZ782902	1	1.8
	RHD*676C	c.676G>C	E 5	p.Ala226Pro	TM7	RHD*16.02	rs3193872	MZ782903	1	1.8
	RHD*329C	c.329T>C	E 2	p.Leu110Pro	TM4	RHD*07.01	rs121912762	MZ782904	1	1.8
	RHD*8G	c.8C> G	E 1	p.Ser3Cys	IC1	RHD*01W.3	rs144969459	MZ782905	1	1.8
	RHD*208T	c.208C>T	E 2	p.Arg70Trp	TM2	RHD* 01W.122	rs542542420	MZ782906	1	1.8
	RHD*18A	c.18G>A	E 1	p.Pro6Pro	IC1	*None^§^*	rs752685469	MZ782907	1	1.8
	RHD*95A	c.95C>A	E 1	p.Thr32Asn	EF1	*None^§^*	rs764093565	MZ782908	1	1.8
	RHD*143G	c.143A>G	E 1	p.Tyr 48Cys	EF1	*/*	/	MZ782891	1	1.8
	RHD*782T#	c.782C>T	E 5	p.Pro261Leu	TM8	*/*	/	MN756604	1	1.8
	RHD*538C#	c.538G>C	E4	p.Gly180Arg	TM6	*/*	/	MN756603	1	1.8
RHD allele with MNV	RHD*DFR1	c.505A>C; c.509T>G; c.514A>T	E 4	p.Met169Leu; p.Met170Arg; p.Ile172Phe	TM6 TM6 TM6	RHD*17.01	rs17421137 / rs17421151	MZ782909	1	1.8
Small insertions	RHD*210_211insG^#^	c.210_211insG	E 2	p.Arg71Glu, 158Ter	TM2	*/*	/	MN756602	1	1.8
RHD-CE-D hybrid allele	RHD*DVI.3	D-CE(3-6)-D	E3-E6	Hybrid	/	RHD*06.03.01	/	MZ782910	14	24.6
	RHD*D-CE(9)-D	D-CE(9)-D	E9	Hybrid	/	*/*	/	MN781673	1	1.8
	RHD*DIIIb	D-CE(2)-D	E2	Hybrid	/	RHD*03.02	/	MZ782911	1	1.8
	RHD*DIIIc	D-CE(3)-D	E3	Hybrid	/	RHD*03.03	/	MZ782912	1	1.8
	RHD*D-CE(7)-D	D-CE(7)-D	E7	Hybrid	/	RHD*58	/	MZ782913	1	1.8
	RHD*D-CE(3-9)-D	D-CE(3-9)-D	E3-E9	Hybrid	/	RHD*01N.04	/	MZ782914	2	3.5

^#^The RHD alleles are the novel mutation sites identified in this study.

^†^Three types of membrane localization of amino acid substitution, IC (intracellular), EF (exofacial), and TM (transmembraneous), were predicted according to the model for orientation of the RhD protein proposed by Wagner and coworkers (25).

^§^These three mutation sites could be found in the SNP database of NCBI, but they were not nominated by ISBT. / They could not be correspond to membrane localization or have no corresponding ISBT terminology and rs numbers.

Two novel single-nucleotide missense variants (c.538G>C and c.782C>T) and one novel insertion variant (c.210_211insG) were detected in three specimens (**Table 2**). All novel *RHD* alleles were found to be hemizygous. RHD*538C allele was consistent with a c.538G>C transition in Exon4 encoding a p.G180R substitution, whereas RHD*782T was associated with a c.782C>T transition in Exon5, resulting in a p.P261L substitution. Both of them corresponded to amino acid substitution in TM. RHD*210_211 insG inserted a G in 210 to 211 positions, resulting in a frameshift to create a premature terminal codon at 158 position of RhD protein.

### Genotyping and Copy Number Analysis Using RH-MLPA Assay

Further genotyping and copy number analysis of 49 RhD variants were performed using MLPA assay (excluding three specimens with no available DNA). The results compared to PCR-SBT are summarized in **Table 1**. Some specimens with commonly known alleles were accurately identified among the tested cases, consistent with PCR-SBT results. RHD*01N.04 alleles were found in two specimens using the RH-MLPA assay but were missing using the PCR-SBT method (**Table 2**). MLPA assay was unable to detect specimens with rare variants (including novel mutation). However, the RhD blood group continues to face the problem of copy number variation. The RH-MLPA assay has a good advantage in analyzing copy number of alleles. Based on the signal ratio of wild-type alleles, nine specimens were found with two copies. The remaining 40 specimens were analyzed for only one copy of *RHD* allele, indicating that they were hemizygous (*RHD/–*). The copy number analysis results based on the MLPA assay were consistent with the zygosity test except for one specimen (RHD*03.02).

### Model 3D Structure of RhD Mutant Protein

**Figure 2:**
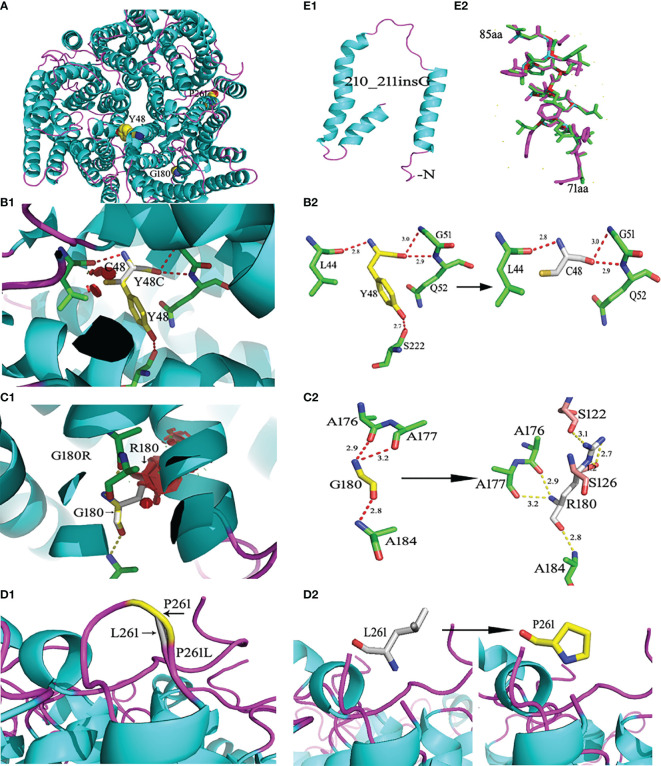
Ribbon diagrams of the wild-type and mutant RhD proteins. RhD proteins were generated from the crystal structure of the RhCG protein (Protein Data Bank accession code 3HD6) template by the SWISS Model. **(A)** Structures of the wild-type RhD and the amino acid residue position with the point mutation. **(B1)** The p.Y48C mutant RhD proteins. **(B2)** The side chain comparison of wild-type Y48 and mutant C48. The dotted line indicates the hydrogen bonds between the amino acid. The number represents the length of the hydrogen bond. **(C1)** The p.G180R mutant RhD proteins. **(C2)** The side chain comparison of wild-type G180 and mutant R180. **(D1)** The loop structural comparison of wild-type RhD and mutant p.P261L protein. **(D2)** The amino acid P261 and substitution L261 were shown by stick. **(E1)** The C-terminally truncated RhD protein caused by c.210_211insG. **(E2)** Fourteen frameshift amino acids from 71 to 85 positions. The purple-red sticks represent the amino acids in the wild-type Rh protein, and the green sticks represent the amino acids in the mutant protein. The mutations were generated using PyMOL software.

The wild-type RhD protein structure was simulated using SWISS-MODEL based on the crystal structure of the RhCG protein model. The amino acid conversion was performed using PyMOL software. The amino acid substitutions in the overall structure of RhD protein are displayed in **Figure 2A**. A known mutation c.143A>G resulted in the substitution of p.Y48C (26), and two novel missense mutations led to p.G180R, and p.P261L substitutions were selected for 3D structure analysis. These three amino acid substitutions were located in α2 helix, α6 helix, and loop ICL3. The connections between the replaced amino acid and other amino acids, as well as the changes in hydrogen bonds, are depicted in **Figures 2B1–D1** and **Table 3**. In the wild-type RhD protein, amino acid residue Y48 was bonded to four amino acids by hydrogens (L44, 2.8Å; G51, 3.0 Å; Q52, 2.9 Å, and S222, 2.7 Å), as indicated in **Figure 2B2**. Following mutagenesis, the hydrogen bond connected to S222 disappeared (**Figure 2B2**). As for p.G180R mutant protein, atoms and positions for forming hydrogen bonds were changed significantly (**Figure 2C2**). Two additional Ser located in α4 helix were bound to R180 (S122, 3.1 Å; S126, 2.7 Å, and 1.2 Å) because Arg has one more side chain than Gly. The hydrogen bond length remained unchanged for the remaining three connected unchanged amino acids (A176, A177, and A184) (**Table 3**). The amino acid at position 261 was located in the intracellular loop. This residue has no interaction with other amino acids analyzed by PyMOL software. **Figures 2D1–D2** demonstrated that the loop’s position was slightly changed because of mutation p.P261L. For RHD*210_211insG allele, a frameshift occurred, resulting in the formation of C-terminally truncated RhD protein, which only formed two complete α-helices (**Figure 2E1**). The model comprised 81 amino acids to the wild-type protein, including 14 frameshift amino acids from 71 to 85 positions. **Figure 2E2** depicted the last 14 different amino acids.

**Table 3:** The parameter changes analysis of the substituted amino acids caused by the non-synonymous mutation in protein 3D structure.

Allele	Wild type				Novel variant allele			
	Amino acid	Predicted no. of hydrogen bonds	Binding amino acids	Hydrogen bond length between amino acid interactions (Å)	Amino acid	Predicted no. of hydrogen bonds	Binding amino acids	Hydrogen bond length between amino acid interactions (Å)
RHD* 143G	Y48	4	L44	2.8	C48	3	L44	2.8
			G51	3.0			G51	3.0
			Q52	2.9			Q52	2.9
			S222	2.7				
RHD* 538C	G180	3	A176	2.9	R180	6	A176	2.9
			A177	3.2			A177	3.2
							S122	3.1
							S126	2.7
								1.2
RHD*782T	P261	0	/	/	L261	0	/	/

/no binding amino acid.

### Predicted Effect of Non-Synonymous Mutations *In Silico*

Three bioinformatics software packages (SIFT, PolyPhen-2, and PROVEAN) were used to predict the influence of non-synonymous mutations on structural alterations. According to deleterious qualification standards, at least two of the three bioinformatics systems utilized had to predict the mutation alleles as damaging. A total of 14 non-synonymous mutations were determined to be deleterious to the protein structure, whereas the remaining mutations were found to be neutral (**Table 4**). The novel non-synonymous mutations were predicted to be deleterious to the protein.

**Table 4:** Predicted effect on RhD protein structure based on nucleotide variation *in silico* using different tools.

Allele name	Nucleotide change	SIFT*	PROVEAN^†^	PolyPhen-2^‡^	Prediction^§^
RHD*Weak partial Type 15	p.G282D	0.001	−6.08	0.929	Deleterious
RHD*101G	p.Y34C	0.001	−6.68	0.995	Deleterious
RHD*weak D type 33	p.V174M	0.058	−1.6	0.316	Neutral
RHD*weak D type 54	p.S122L	0.01	−3.58	0.44	Deleterious
RHD*weak D type 36	p.V281G	0	−6.23	0.862	Deleterious
RHD*weak D type 25	p.R114Q	0.625	0	0.019	Neutral
RHD*weak D type 85	p.R70Q	0.044	−2.29	0.248	Neutral
RHD*weak D type 47	p.R114G	0.438	−2.5	0.499	Deleterious
RHD*weak D type 100	p.G263R	0.146	−6.4	0.525	Deleterious
RHD*weak D type 72	p.N404E	0	−3.07	1.000	Deleterious
RHD*DFR1	p.M169L	0.378	−1.22	0.002	Neutral
	p.M170R;	0.168	−4.07	0.032	Neutral
	p.I172F	0.261	−2.38	0.01	Neutral
RHD*DCS2	p.A226P	0.095	−3.18	0.019	Neutral
RHD*DVII.1	p.L110P	0.033	−5.04	0.997	Deleterious
RHD*weak D type 3	p.S3C	0.023	−3.45	0.995	Deleterious
RHD*weak D type 122	p.R70W	0.064	−4.63	0.159	Neutral
RHD*95A	p.T32N	0.013	−3.1	0.913	Deleterious
RHD*1022A	p. I341N	0.001	−4.6	0.845	Deleterious
RHD*782T	p.P261L	0.006	−5.99	0.969	Deleterious
RHD*538C	p.G180R	0.000	−7.13	1.000	Deleterious
RHD*143G	P.Y48C	0.000	−7.011	1.000	Deleterious

*SIFT, Sorting Intolerant From Tolerant; score ≤0.05 = damaging, >0.05 = tolerated.

^†^PROVEAN, Protein Variation Effect Analyzer; score >−2.5 = neutral, ≤−2.5 = deleterious.

^‡^PolyPhen-2, Polymorphism Phenotypingv2; score 0.000–0.452 = benign, 0.453–0.956 = possibly damaging, 0.957–1.000 = probably damaging.

^§^To qualify as deleterious, the mutation had to be predicted as damaging by at least two of the three bioinformatics programs used.

## Discussion

In this study, the molecular characteristics of RhD variants were investigated in Zhejiang Province, eastern China. Genotyping is extensively applied to identify RhD variants (27). As the most important and sophisticated clinical blood group system, serological testing is hampered by many factors and cannot clearly identify various RhD variants. Notably, distinct phenotypes and genotypes of RhD variants have specific clinical significance and require blood transfusion strategies (28, 29). The RhD blood type mismatch between donor and recipient may cause acute severe immune response resulting in neonatal hemolytic disease, hemolytic transfusion reaction, and autoimmune hemolytic disease (26, 30). Therefore, genotyping is required to determine the molecular characteristics of RhD variant donors or recipients, which is beneficial to disease treatment. As an alternative to routine serological testing, genotyping has a significant potential function in clinical practice.

*RHD* allele distribution varies widely between ethnic populations. At present, 28 distinct variant *RHD* alleles, including three novel alleles, were characterized in our tested specimens. RHD*DVI.3 and RHD*weak partial 15 were the most prevalent RhD variant alleles in Zhejiang province. This result was consistent with the distribution of RhD variant alleles in other regions of China (9, 16, 31), but it is completely different from a Caucasian population with a prevalence of less than 5% (32, 35). There was no significant similarity or clustering in a particular site between the other rare RhD variant alleles identified in our study and those reported previously (3, 9). In this study, the frequencies of variant *RHD* alleles could not be accurately calculated because the specimens were not derived from a unified large-scale screening of random populations. However, their frequency was definitely low, with only one to two specimens detected in the population.

This study identified three novel D variant alleles, which provided knowledge of *RHD* alleles and enriched the genetic resources of Rh blood group in the Chinese population. The c.538G>C mutation is similar to the c.538G>A mutation reported by Fichou et al. (12), since they have different base mutations at the same position, causing the same amino acid substitution. The novel RHD*210_211insG allele predicting a premature terminal codon was derived from the pregnant woman with the RhD-negative phenotype. However, the RhD-negative phenotype required confirmation using absorption and elution test. Subsequent corresponding expression experiments *in vitro* are required to elucidate the effect of mutation on the antigen.

The Del caused by RHD*1227A holds a significant function in the Asian population, which is referred to as “Asian Del”. In clinical practice, RhD-negative individuals were confirmed to produce primary and secondary immune responses caused by Asian Del transfusion (33, 34). On the other hand, pregnant women with “Asian Del” would not produce IgG antibodies to cause hemolytic disease of newborn (HDN) (35), indicating that *RHD* genotyping could avoid unnecessary administration of RhIg. Notably, some variants may have a strong immune response. The variant arising from RHD*D-CE(9)-D was reported to produce a high-titer anti-D and cause severe hemolytic disease in newborns (36). To improve blood transfusion safety and early prevention of Rh-related neonatal hemolytic disease, it is required to broaden the use of Rh blood group genotyping, including RhD-negative individuals, which could reduce immunizations caused by antibodies. RhD genotyping has been widely implemented in European countries to control blood product supply (37–39).

All mutation sites appeared to be randomly distributed over the CDS region. The variant alleles affecting transmembrane region were relatively prevalent among these rare RhD variant alleles. The possible reason for the impact of these variant alleles on antigens is yet unclear because different variant alleles exhibit distinct phenotypic and clinical characteristics (25, 28). As a result, knowledge of *RHD* alleles and their clinical significance contributed to each case and the development of blood group genetic factors. In China and other populations, much more practical research on the Rh blood group is encouraged.

The difficulty in investigating the molecular mechanism of Rh blood group is not only in the sequence analysis but also in identifying *RHD-CE* recombination and *RH* gene copy number variation (40). However, there are few accurate detection methods available in this area. The copy number of *RHD* gene directly affects the expression of its surface antigen, which is greatly significant for prenatal diagnosis and prevention of hemolytic disease of fetus and newborn (HDFN) (41). Therefore, detecting *RHD* gene copy number is critical. At present, the MLPA assay and hybridization box test are relatively suitable methods for heterozygosity detection. In this study, the MLPA method was employed to concurrently perform genotyping and copy number variation analysis for RhD variants. However, the results were inconsistent with hybrid Rhesus test for one specimen because hybrid Rhesus detection may be affected by differences in breakpoints or additional variations in Rhesus box area (42, 43). In terms of detecting point mutations, the MLPA assay is weaker than PCR-SBT, particularly for weak D variants. It cannot detect most rare mutation sites and is even more challenging to detect novel mutations because it contains only a few well-established mutation sites. On the other hand, the PCR-SBT method cannot determine copy number variation of *RHD* gene, and hybridization box PCR-SSP test has a relatively high error rate (43). Therefore, these three technologies have their own advantages and disadvantages and complement each other. To achieve different clinical requirements, combining the actual needs and choosing the appropriate method for Rh blood group genotype is recommended.

According to 3D simulated protein structure, amino acid changes caused by missense mutations, p.Y48C, and p.G180R affect the secondary structure α-helix of protein. Tyr and Cys are polar hydrophilic amino acids; however, p.Y48C substitution eliminated the aromatic ring of amino acids and reduced hydrogen bonds connected to S222 in another α-helix. Therefore, replacing this amino acid may disrupt the interaction between α-helix structures, resulting in decreased antigen expression or changes in antigenic properties. G180 is the smallest hydrophobic amino acid for a wild type, making it a superior helix-forming residue (44). Compared with Gly, Arg has a large and hydrophilic charged side chain, impacting tertiary interactions and RhD protein stabilization. Meanwhile, hydrogen bonds were affected by the side-chain group. G180R substitution increased the interaction with other amino acids by adding three hydrogen bonds. However, additional hydrogen bonds may not be required for correct helix folding. Instead, it may destroy the inherent stable structure of protein and impair its membrane insertion (45). The reduction of RhD antigen density caused by p.P261L substitution may be explained by the disruptive effect of Leu interfering with correct folding of intracellular loop ICL3. The insertion mutation RHD*210_211insG caused a frameshift at amino acid 71 and premature termination, resulting in a truncated RhD polypeptide. As a result, it was speculated that this insertion could only comprise a part of RhD epitope. The 3D structural simulation revealed that protein’s spatial conformation was destroyed. Amino acid substitutions altered the interaction and stability of RhD protein structure, whereas frameshift mutations resulted in partial structure loss. All of them affected the normal assembly of tertiary structure, leading to changes in RhD antigen characteristics. The bioinformatics analysis of RhD protein could help us grasp the impact of *RHD* gene mutations on antigen differences and guide subsequent blood transfusion strategies.

Seven specimens defined as D variants could not detect corresponding variant alleles and may have another molecular basis. RHD-specific microRNA was reported to regulate the expression of Rh antigen protein (46). However, the molecular mechanism underlying the differential expression of RhD antigen remains largely unknown. Additional research is required to determine the impact of other regulatory elements on RhD variants.

In summary, this study identified and characterized various *RHD* alleles, including 25 reported weak D, partial D, DEL, RhD alleles, and three novel variant alleles. These findings provided a brief overview of variant D phenotypes found in eastern China. Additionally, we performed an *in silico* analysis of bioinformatics of these variant alleles. All findings could extend our knowledge of RhD variants in blood donors and clinical transfusion recipients. It is critical for precision blood transfusion treatment, as it reduces the risk of alloimmunization in RhD-negative recipients to improve blood transfusion safety, organ transplantation success, and fetal incompatibility prevention.

## Data Availability Statement

The datasets presented in this study can be found in online repositories. The names of the repository/repositories and accession number(s) can be found below: GenBank, accession numbers from MZ782891 to MZ782914.

## Ethics Statement

The studies involving human participants were reviewed and approved by the Ethics committee of Blood Center of Zhejiang Province. The patients/participants provided their written informed consent to participate in this study.

## Author Contributions

YY performed research, analyzed data, and wrote the paper. JZ, XH, and XX performed research. JH collected the samples. FZ designed the research. All authors contributed to the article and approved the submitted version.

## Conflict of Interest

The authors declare that the research was conducted in the absence of any commercial or financial relationships that could be construed as a potential conflict of interest.

## Publisher’s Note

All claims expressed in this article are solely those of the authors and do not necessarily represent those of their affiliated organizations, or those of the publisher, the editors and the reviewers. Any product that may be evaluated in this article, or claim that may be made by its manufacturer, is not guaranteed or endorsed by the publisher.

## References

[1] Dinardo CL Kelly S Dezan MR Ribeiro IH Castilho SL Schimidt LC. NHLBI Recipient Epidemiology and Donor Evaluation Study (REDS)-III. Diversity of RH and Transfusion Support in Brazilian Sickle Cell Disease Patients With Unexplained Rh Antibodies. Transfusion (2019) 59(10):3228–35. doi: 10.1111/trf.15479 PMC6785370 31408202

[2] Sahoo T Sahoo M Gulla KM Gupta M. Rh Alloimmunisation: Current Updates in Antenatal and Postnatal Management. Indian J Pediatr (2020) 87(12):1018–28. doi: 10.1007/s12098-020-03366-0 32607667

[3] Flegel WA. Molecular Genetics and Clinical Applications for RH. Transfus Apher Sci (2011) 44(1):81–91. doi: 10.1016/j.transci.2010.12.013 21277262 PMC3042511

[4] Shi J Luo Y. Effects of RHD Gene Polymorphisms on Distinguishing Weak D or DEL From RhD- in Blood Donation in a Chinese Population. Mol Genet Genomic Med (2019) 7(6):e00681. doi: 10.1002/mgg3.681 30950221 PMC6565595

[5] Trucco Boggione C Nogués N González-Santesteban C Mufarrege N Luján Brajovich M Mattaloni SM. Characterization of RHD Locus Polymorphism in D Negative and D Variant Donors From Northwestern Argentina. Transfusion (2019) 59(10):3236–42. doi: 10.1111/trf.15504 31503349

[6] Sandler SG Chen LN Flegel WA. Serological Weak D Phenotypes: A Review and Guidance for Interpreting the RhD Blood Type Using the RHD Genotype. Br J Haematol (2017) 179(1):10–9. doi: 10.1111/bjh.14757 PMC5612847 28508413

[7] Srivastava K Polin H Sheldon SL Wagner FF Grabmer C Gabriel C. The DAU Cluster: A Comparative Analysis of 18 RHD Alleles, Some Forming Partial D Antigens. Transfusion (2016) 56(10):2520–31. doi: 10.1111/trf.13739 PMC5499517 27480171

[8] de Paula Vendrame TA Prisco Arnoni C Guilhem Muniz J de Medeiros Person R Pereira Cortez AJ Roche Moreira Latini F. Characterization of RHD Alleles Present in Serologically RHD-Negative Donors Determined by a Sensitive Microplate Technique. Vox Sang (2019) 114(8):869–75. doi: 10.1111/vox.12851 31587310

[9] Zhang X Li G Zhou Z Shao C Huang X Li L. Molecular and Computational Analysis of 45 Samples With a Serologic Weak D Phenotype Detected Among 132,479 Blood Donors in Northeast China. J Transl Med (2019) 27 17(1):393. doi: 10.1186/s12967-019-02134-9 PMC6880393 31775789

[10] Stef M Fennell K Apraiz I Arteta D González C Nogués N. RH Genotyping by Nonspecific Quantitative Next-Generation Sequencing. Transfusion (2020) 60(11):2691–701. doi: 10.1111/trf.16034 32871036

[11] Matteocci A Monge-Ruiz J Stef M Apraiz I Herrera-Del-Val L Mancuso T. Two New RHD Alleles With Deletions Spanning Multiple Exons. Transfusion (2021) 61(3):682–6. doi: 10.1111/trf.16199 33241598

[12] Fichou Y Le Marechal C Bryckaert L Guerry C Benech C Dupont I. Variant Screening of the RHD Gene in a Large Cohort of Subjects With D Phenotype Ambiguity: Report of 17 Novel Rare Alleles. Transfusion (2012) 52:759–64. doi: 10.1111/j.1537-2995.2011.03350.x 21950494

[13] Cruz BR Chiba AK Moritz E. RHD Alleles in Brazilian Blood Donors With Weak D or D-Negative Phenotypes. Transfus Med (2012) 22:84–9. doi: 10.1111/j.1365-3148.2011.01129.x 22211984

[14] Tounsi WA Madgett TE Avent ND. Complete RHD Next-Generation Sequencing: Establishment of Reference RHD Alleles. Blood Adv (2018) 2(20):2713–23. doi: 10.1182/bloodadvances.2018017871 PMC6199663 30337299

[15] Wheeler MM Lannert KW Huston H Fletcher SN Harris S Teramura G. NHLBI Trans-Omics for Precision Medicine (TOPMed) Consortium. Genomic Characterization of the RH Locus Detects Complex and Novel Structural Variation in Multi-Ethnic Cohorts. Genet Med (2019) 21(2):477–86. doi: 10.1038/s41436-018-0074-9 PMC6311147 29955105

[16] He J Ying Y Hong X Xu X Zhu F Lv H. Molecular Basis and Zygosity Determination of D Variants Including Identification of Four Novel Alleles in Chinese Individuals. Transfusion (2015) 55(1):137–43. doi: 10.1111/trf.12797 25070883

[17] Perco P Shao CP Mayr WR Panzer S Legler TJ. Testing for the D Zygosity With Three Different Methods Revealed Altered Rhesus Boxes and a New Weak D Type. Transfusion (2003) 43(3):335–9. doi: 10.1046/j.1537-2995.2003.00313.x 12675718

[18] Kim B Lee ST Kim S Choi JR Kim HO. Application of Multiplex Ligation-Dependent Probe Amplification Assay for Genotyping Major Blood Group Systems Including DEL Variants in the D-Negative Korean Population. Ann Lab Med (2018) 38(1):32–8. doi: 10.3343/alm.2018.38.1.32 PMC5700144 29071816

[19] Gruswitz F Chaudhary S Ho JD Schlessinger A Pezeshki B Ho C-M. Function of Human Rh Based on Structure of RhCG at 2.1 Å. Proc Natl Acad Sci U SA (2010) 107:9638–43. doi: 10.1073/pnas.1003587107 PMC2906887 20457942

[20] Arnold K Bordoli L Kopp J Schwede T. The SWISS-MODEL Workspace: A Web-Based Environment for Protein Structure Homology Modelling. Bioinformatics (2006) 22:195–201. doi: 10.1093/bioinformatics/bti770 16301204

[21] Rigsby RE Parker AB. Using the PyMOL Application to Reinforce Visual Understanding of Protein Structure. Biochem Mol Biol Educ (2016) 44(5):433–7. doi: 10.1002/bmb.20966 27241834

[22] Adzhubei IA Schmidt S Peshkin L Ramensky VE Gerasimova A Bork P. A Method and Server for Predicting Damaging Missense Mutations. Nat Methods (2010) 7:248–9. doi: 10.1038/nmeth0410-248 PMC2855889 20354512

[23] Ng PC Henikoff S. Predicting Deleterious Amino Acid Substitutions. Genome Res (2001) 11:863–74. doi: 10.1101/gr.176601 PMC311071 11337480

[24] Choi Y Sims GE Murphy S Miller JR Chan AP. Predicting the Functional Effect of Amino Acid Substitutions and Indels. PloS One (2012) 7:e46688. doi: 10.1371/journal.pone.0046688 23056405 PMC3466303

[25] Wagner FF Gassner C Müller TH. Molecular Basis of Weak D Phenotypes. Blood (1999) 93:385–93. doi: 10.1182/blood.V93.1.385 9864185

[26] Filosa L Beley S Chiaroni J Bailly P Silvy M. New Silent and Weak D Alleles: Molecular Characterization and Associated Antigen Density. Transfusion (2016) 56(8):2154–5. doi: 10.1111/trf.13655 27189905

[27] Westhoff CM. Blood Group Genotyping. Blood (2019) 133(17):1814–20. doi: 10.1182/blood-2018-11-833954 30808639

[28] Sreelekshmi S Shastry S Poornima Baliga B. Variable Reactivity of Rh D Antigen and its Serological Characterization. Acta Clin Belg (2021) 76(5):346–50. doi: 10.1080/17843286.2020.1735115 32108563

[29] Sippert E Fujita CR Machado D Guelsin G Gaspardi AC Pellegrino J Jr. Variant RH Alleles and Rh Immunisation in Patients With Sickle Cell Disease. Blood Transfus (2015) 13(1):72–7. 10.2450/2014.0324-13 PMC4317093 24960646

[30] van der Schoot CE de Haas M Clausen FB. Genotyping to Prevent Rh Disease: Has the Time Come? Curr Opin Hematol (2017) 24(6):544–50. doi: 10.1097/MOH.0000000000000379 28937404

[31] Chen Q Li M Li M Lu XS Lü R Sun J. Molecular Basis of Weak D and DEL in Han Population in Anhui Province, China. Chin Med J (Engl) (2012) 125(18):3251–5. 22964318

[32] Flegel WA von Zabern I Doescher A. D Variants at the RhD Vestibule in the Weak D Type 4 and Eurasian D Clusters. Transfusion (2009) 49:1059–69. doi: 10.1111/j.1537-2995.2009.02102.x PMC10710224 19309476

[33] Shao CP. Transfusion of RhD-Positive Blood in “Asia Type” DEL Recipients. N Engl J Med (2010) 362(5):472–3. doi: 10.1056/NEJMc0909552 20130261

[34] Yang HS Lee MY Park TS Cho SY Lee HJ Lim G. Primary Anti-D Alloimmunization Induced by “Asian Type” RHD (C.1227G>A) DEL Red Cell Transfusion. Ann Lab Med (2015) 35(5):554–6. doi: 10.3343/alm.2015.35.5.554 PMC4510514 26206698

[35] Shao CP Xu H Xu Q Sun GD Li JP Zhang BW. Antenatal Rh Prophylaxis is Unnecessary for “Asia Type” DEL Women. Transfus Clin Biol (2010) 17(4):260–4. doi: 10.1016/j.tracli.2010.07.003 20961786

[36] Jakobsen MA Nielsen C Sprogøe U. A Case of High-Titer Anti-D Hemolytic Disease of the Newborn in Which Late Onset and Mild Course is Associated With the D Variant, RHD-CE(9)-D. Transfusion (2014) 54(10):2463–7. doi: 10.1111/trf.12673 24749928

[37] de Haas M Thurik FF van der Ploeg CP. Sensitivity of Fetal RHD Screening for Safe Guidance of Targeted Anti-D Immunoglobulin Prophylaxis: Prospective Cohort Study of a Nationwide Programme in the Netherlands. BMJ (2016) 355:i5789. doi: 10.1136/bmj.i5789 27821701 PMC5098549

[38] Soothill PW Finning K Latham T Wreford Bush T Ford J Daniels G. Use of cffDNA to Avoid Administration of Anti-D to Pregnant Women When the Fetus is RhD-Negative: Implementation in the NHS. BJOG (2015) 122(12):1682–6. doi: 10.1111/1471-0528.13055 25142171

[39] Clausen FB Rieneck K Krog GR Bundgaard BS Dziegiel MH. Noninvasive Antenatal Screening for Fetal RHD in RhD Negative Women to Guide Targeted Anti-D Prophylaxis. Methods Mol Biol (2019) 1885:347–59. doi: 10.1007/978-1-4939-8889-1_23 30506209

[40] Haer-Wigman L Veldhuisen B Jonkers R. RHD and RHCE Variant and Zygosity Genotyping *via* Multiplex Ligation–Dependent Probe Amplification. Transfusion (2013) 53:1559–74. doi: 10.1111/j.1537-2995.2012.03919.x 23043317

[41] Pirelli KJ Pietz BC Johnson ST Pinder HL Bellissimo DB. Molecular Determination of RHD Zygosity: Predicting Risk of Hemolytic Disease of the Fetus and Newborn Related to Anti-D. Prenat Diagn (2010) 30(12-13):1207–12. doi: 10.1002/pd.2652 21072752

[42] Wagner FF Moulds JM Flegel WA. Genetic Mechanisms of Rhesus Box Variation. Transfusion (2005) 45:338–44. doi: 10.1111/j.1537-2995.2005.04339.x 15752150

[43] Grootkerk-Tax MG Maaskant-van Wijk PA van Drunen J. The Highly Variable RH Locus in Nonwhite Persons Hampers RHD Zygosity Determination But Yields More Insight Into RH-Related Evolutionary Events. Transfusion (2005) 45:327–37. doi: 10.1111/j.1537-2995.2005.04199.x 15752149

[44] Javadpour MM Eilers M Groesbeek M Smith SO. Helix Packing in Polytopic Membrane Proteins: Role of Glycine in Transmembrane Helix Association. Biophys J (1999) 77:1609–18. doi: 10.1016/S0006-3495(99)77009-8 PMC1300449 10465772

[45] Silvy M Chapel-Fernandes S Callebaut I Beley S Durousseau C Simon S. Characterization of Novel RHD Alleles: Relationship Between Phenotype, Genotype, and Trimeric Architecture. Transfusion (2012) 52(9):2020–9. doi: 10.1111/j.1537-2995.2011.03544.x 22320258

[46] Thongbut J Kerdpin U Sakuldamrongpanich T Isarankura Na-Ayudhya C Nuchnoi P. RHD-Specific microRNA for Regulation of the DEL Blood Group: Integration of Computational and Experimental Approaches. Br J BioMed Sci (2017) 74(4):181–6. doi: 10.1080/09674845.2017.1331522 28730912

